# Low Turnover of Soil Bacterial rRNA at Low Temperatures

**DOI:** 10.3389/fmicb.2020.00962

**Published:** 2020-05-25

**Authors:** Morten Dencker Schostag, Christian Nyrop Albers, Carsten Suhr Jacobsen, Anders Priemé

**Affiliations:** ^1^Department of Geosciences and Natural Resource Management, Center for Permafrost (CENPERM), University of Copenhagen, Copenhagen, Denmark; ^2^Department of Biology, University of Copenhagen, Copenhagen, Denmark; ^3^Geological Survey of Denmark and Greenland, Copenhagen, Denmark; ^4^Department of Environmental Science, Aarhus University, Roskilde, Denmark

**Keywords:** ribosomal RNA, rRNA half-life, environmental RNA, uridine, temperature response

## Abstract

Ribosomal RNA (rRNA) is used widely to investigate potentially active microorganisms in environmental samples, including soil microorganisms and other microbial communities that are subjected to pronounced seasonal variation in temperature. This raises a question about the turnover of intracellular microbial rRNA at environmentally relevant temperatures. We analyzed the turnover at four temperatures of RNA isolated from soil bacteria amended with ^14^C-labeled uridine. We found that the half-life of recently produced RNA increased from 4.0 days at 20°C to 15.8 days at 4°C, and 215 days at −4°C, while no degradation was detected at −18°C during a 1-year period. We discuss the implications of the strong temperature dependency of rRNA turnover for interpretation of microbiome data based on rRNA isolated from environmental samples.

## Introduction

Microbial DNA isolated from environmental samples cannot differentiate between the live and dead fraction of the microbiome and is generally believed to persist for longer time spans compared to RNA, e.g., in soils where extracellular DNA from dead organisms may persist for years (Carini et al., 2016). The persistence of DNA in the environment obscures relationships between microbiomes and environmental parameters. Hence, analysis of the more dynamic RNA instead of DNA has over the last 10 years received increased attention in studies of e.g., the response of microbiomes to global change and environmental perturbation like thawing of frozen soil (Schostag et al., 2019), wetting of dry soil (Placella et al., 2012), and xenobiotics (Baelum et al., 2008). Ribosomal RNA (rRNA) is now widely used in microbiome studies to characterize the phylogenetic composition of potentially active members of microbial communities [see Blazewicz et al. (2013) for a discussion of the interpretation of rRNA in an ecological context]. The large interest in rRNA from environmental microbiomes contrasts the lack of knowledge on the turnover of microbial rRNA in the environment.

Bacterial rRNA is more stable than messenger RNA (mRNA) likely as a consequence of its incorporation into ribosomes and protection by ribosomal proteins (Deutscher, 2003), but rRNA is degraded by RNases intracellularly during stress, e.g., caused by starvation for organic carbon, amino acids or essential nutrients (Zundel et al., 2009), or when an RNA molecule is defective (Deutscher, 2006). Bacterial ribosomes may also be inactivated and maintained as such under no-growth conditions (Dai et al., 2016). Numerous studies and commercial laboratory suppliers have described the decay of RNA in bacterial strains or in environmental samples under different conditions (often in combination with substances that inhibit RNase activity), but these studies focus on the decay of mRNA or the total pool of RNA that also includes extracellular RNA. Degradation of extracellular rRNA likely depends on environmental conditions as temperature, water availability, pH [RNA can undergo auto-hydrolysis at high pH when free hydroxide ions deprotonate the hydroxyl-group on the C2 of the ribose moiety (Elliot and Ladomery, 2011)], and soil mineralogy [RNA may sorb to soil minerals as known for DNA (Romanowski et al., 1991)]. To our knowledge, the present study is the first to estimate turnover of intracellular rRNA in an environmental microbiome at different temperatures.

## Materials and Methods

Studies of RNA turnover in bacterial cells often involve inhibition of RNA synthesis by actinomycin D (Levinthal et al., 1962) or rifampicin (Hambraeus et al., 2003). In contrast, we developed an experimental approach, which did not inhibit the general activity of the bacteria. In brief, we extracted bacterial cells from soil, enhanced their production of RNA by adding diluted tryptic soy broth (TSB) growth medium before adding ^14^C-radiolabeled uridine (an RNA nucleoside), and finally returning the bacteria to the soil for incubation at −18, −4, 4, and 20°C, respectively. At different time points, RNA was isolated from the incubated soil samples and its content of ^14^C was measured.

### Soil

Soil was sampled in April 2016 at 0–10 cm depth from a permanent grassland at the University of Copenhagen experimental farm Højbakkegård, Tåstrup, Denmark. The mean annual air temperature is 8.2°C. The soil is a sandy loam with pH 6.4 and had a moisture content of 22.7% of dry weight at the time of sampling. The soil was gently homogenized by hand and sieved at 4 mm.

### Extraction of Bacteria From Soil Matrix, Pre-incubation of Bacteria, and Amendment With ^14^C-Uridine

Two batches of 100 g soil in 320 mL phosphate-buffered saline (PBS) were blended in a sterilized blender for 3 min. An additional 80 mL PBS was used to wash away remaining soil particles after transferring the blended soil slurry to eight 50-mL tubes, which were centrifuged at 1000*g* for 5 min at 4°C. Thirty mL of the supernatant and 60 mL 1-% TSB were transferred to eight incubation flasks for a final concentration of 1/150 TSB. The flasks were incubated at 4°C with shaking at 150 rpm. After 24 h, TSB was added to a final concentration of 1/75, and following an additional 24 h, TSB was added to a final concentration of 1/10 and the flasks were incubated for 24 h. A pilot experiment revealed that this gradual increase in TSB concentration was needed to achieve adequate incorporation of ^14^C in RNA. The pilot experiment involved incubation of extracted bacteria at three separate concentrations of TSB (1/150, 1/75, and 1/10 TSB), and scintillation counting was conducted after 3 days of incubation to quantify the incorporation of ^14^C into cells following amendment with ^14^C-radiolabeled uridine (2-^14^C-uridine, 53 mCi mmol^–1^, purity 98.7%, Moravek, Brea, CA, United States). Twenty hours after addition of labeled uridine, the remaining radioactivity in the media was 11.2, 14.2, and 91.5% of the total added following incubation at 1/150, 1/75, and 1/10 TSB, respectively, and after centrifugation, 5.4% of the radioactivity was found in cells incubated with 1/150 TSB, 6.4% for 1/75 TSB, and 69.5% for 1/10 TSB. The low incorporation of radioactivity by cells incubated with 1/150 and 1/75 TSB was most likely caused by bacteria using the labeled uridine mainly as an energy source resulting in a loss of ^14^C as ^14^CO_2_.

The contents of the incubation flasks were combined and 60 mL were transferred to eight 250-mL flasks and amended with 60 μL of ^14^C-uridine to a final concentration of 6.2 × 10^5^ disintegrations per min (DPM) mL^–1^. Uridine is specifically incorporated into RNA or used as an energy, carbon and/or nitrogen source by the soil microorganisms, but RNA can be selectively isolated from other cell substances that may contain some of the ^14^C.

Following incubation of the eight flasks for 23 h at 4°C at 150 rpm, the content was transferred to fourteen 50-mL tubes and centrifuged at 6000*g* for 5 min at 10°C. The supernatant was decanted and cells were re-suspended in 30 mL PBS by vortexing. The cells were washed twice in 4 mL PBS by centrifuging as described above. 2.5 mL of re-suspended bacteria were mixed using a spatula into 25 g of soil that had been air-dried for 24 h at room temperature. Care was taken to distribute the suspension evenly in the soil. 1.4⋅10^5^ bacterial cells mL^–1^ were transferred as estimated by counting the cells in a fluorescence microscope following acridine orange staining. Only bacterial cells were observed in the microscoped samples.

### RNA Isolation and Calculation of rRNA Half-Life

The soil amended with ^14^C-labeled bacteria was distributed in 2.0-g aliquots to 2-mL plastic tubes from the RNA isolation kit (RNeasy PowerSoil Total RNA Kit, MO BIO Laboratories, Qiagen, Carlsbad, CA, United States), and the soil samples were incubated in our laboratory in quadruplicates at −18°C, −4°C, 4°C, and 20°C, respectively. When sampling for RNA, the tubes were snap frozen in liquid nitrogen and stored at −80°C until RNA isolation using the RNeasy PowerSoil kit. From the final volume of 100 μL eluate, 40 μL was added to 10 mL scintillation liquid in a 20-mL scintillation vial, which was analyzed on a scintillation counter (Tri-Carb 2810 TR, PerkinElmer, Waltham, MA, United States).

A comparison of the radioactivity in the cell suspension (*n* = 2) and RNA isolated from the soil samples at initiation of the experiment (*n* = 15) indicated that the RNA isolation procedure was highly efficient, isolating 97.4% of the labeled RNA pool from the soil samples relative to the cell suspension. As a side note, our experimental setup may, thus, also be used to evaluate the efficiency of protocols for isolation of RNA from soil and other environmental samples.

We tested if ^14^C in the eluate was incorporated in DNA and not as expected in RNA. We did this for the samples incubated at 4 and 20°C, respectively. Two μL DNase-free RNase (Roche Diagnostics, Mannheim, Germany) and 28 μL H_2_O were added to 20 μL eluate from the RNA isolation procedure, and the mix was incubated at 37°C for 1 h. DNA was isolated from the solution using the Genomic DNA Clean & Concentrator kit (Zymo Research, Irvine, CA, United States) according to the manufacturer’s instructions. All flow-through liquid (presumed to contain nucleotides from degraded RNA) from the washing steps was combined and analyzed for radioactivity. The DNA concentration in the eluate (presumed to contain DNA) was quantified using a Qubit Fluorometer (Thermo Fisher Scientific, Hvidovre, Denmark) and the eluate was analyzed for radioactivity. This control experiment revealed that 99.55 ± 0.03% (*n* = 8) of the radioactivity in the eluate from the RNA isolation was found in the RNA fraction and only 0.45 ± 0.03% of the radioactivity was found in the DNA fraction.

As the total RNA pool in the soil may be affected by the experimental treatment we normalized the labeled RNA to the total RNA pool (estimated using Qubit 2.0 [Thermo Fisher Scientific]) at each sampling point (see Supplementary Material). Hereafter, the half-life of the labeled RNA was calculated from the relative normalized DPM assuming an exponential decrease in DPM with time (*r*^2^ ranged from 0.435 to 0.765 for the four replicate samples incubated at −4°C, from 0.926 to 0.982 for the samples at 4°C, and from 0.924 to 0.944 for the samples at 20°C).

## Results

It has previously been shown that approximately 95% of total RNA isolated from soil microbiomes is rRNA and that the majority is bacterial (Tveit et al., 2013; Schostag et al., 2019). We assumed that the mRNA pool has a faster turnover compared to rRNA and that a large proportion of the initial RNA degradation would be due to degradation of mRNA. Because our focus was rRNA we excluded the first measurement of radioactivity, time 0, from the calculation of turnover rates. Also, the last two measurements at 20°C were excluded from the calculations because the radioactivity at these measurements did not follow the same exponential decrease as the previous measurements (at this point less than 10% of the initial radioactivity was found in the RNA pool). We expect that microRNA and small interfering RNA are too small to be collected by the RNA isolation kit. Thus, the reported turnover rates mainly represent turnover of bacterial 16S, 23S, and 5S rRNA, and to an unknown extent transfer RNA.

**FIGURE 1:**
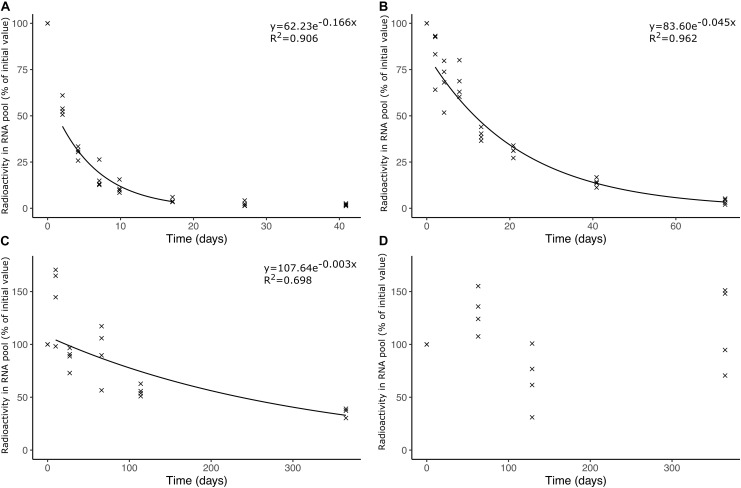
Radioactivity in RNA isolated from soil samples incubated at different temperatures. The samples were amended with soil bacteria pre-incubated with ^14^C-uridine normalized to the total amount of RNA isolated from individual samples. The soil samples were incubated at **(A)** 20°C, **(B)** 4°C, **(C)** –4°C, and **(D)** –18°C. Trend lines in **(A–C)** represent the average radioactivity of replicate samples and depict the time span with exponential decrease in radioactivity presumed to be related to rRNA turnover.

We found a strong temperature dependence of soil bacterial rRNA turnover (Figure 1) with undetectable degradation at −18°C, and a half-life for presumed rRNA of 215 ± 12 days (average ± standard error of the mean, *n* = 4) at −4°C, 15.8 ± 1.00 days at 4°C, and 4.04 ± 0.44 days at 20°C.

## Discussion

Little data exist on the turnover of intracellular rRNA in environmental microbiomes. Keith et al. (2005) found no change in the rRNA-based structure of the bacterial microbiome in activated sludge stored for 24 h at room temperature and only small changes when storing at 4°C, but the study does not report if changes in the amount of RNA occurred. Ostle et al. (2003) reported a turnover of bacterial and archaeal RNA of 20% day^–1^ corresponding to a half-life of 3.1 days in a grassland rhizosphere where plants were spiked with ^13^CO_2_ (at air temperatures of ca. 15°C). This is faster than the turnover at 20°C in our study even though our experimental setup may overestimate the rRNA turnover. This possible overestimation may be due to the transferred bacteria being stimulated by the (albeit dilute) TSB growth medium before transfer to the soil where nutrient availability is expected to be lower, which may lead to starvation of the transferred bacteria and hence enhanced rRNA decay (Zundel et al., 2009). Also, extrinsic factors may increase the turnover of intracellular rRNA, e.g., protozoa may enhance the turnover of bacterial rRNA when grazing the labelled bacteria.

The slow RNA turnover we observed at −4°C is likely due to lack of liquid H_2_O (Tilston et al., 2010) and is in accordance with Schostag et al. (2015) who found no differences in the number of 16S rRNA copies in an Arctic soil microbiome sampled monthly during winter, where the *in situ* temperature was below −4°C for five consecutive months. Thus, the rRNA isolated during winter probably did not reflect sustained microbial activity, but rather was produced by the microbiome during autumn and was conserved for several months due to the low soil temperature. Our lowest incubation temperature, −18°C, preserved bacterial rRNA within the time span of the experiment, which has also been observed by Sessitsch et al. (2002).

The large difference in half-life observed at −4 and 4°C is likely caused by not only temperature *per se*, but also by the different states of soil H_2_O (ice and water, respectively) prevailing at the two temperatures. Ice lowers diffusion rates of nutrients and gases dramatically in the soil environment (Tilston et al., 2010) and, hence, strongly limits soil microbial activity. The 3.9-fold difference in half-life at 4 and 20°C indicates a Q_10_ of 2.3 for this temperature span. However, the Q_10_ value is based on only two temperatures and more detailed studies are needed to generate robust temperature response curves of the bacterial rRNA half-life in soil and other environments.

Pronounced seasonal variation in environmental temperature is part of life for microorganisms living in soil, shallow-water sediments, surface water, poikilothermic animals, or on plants. Our data indicate that half of the intracellular rRNA isolated from an environmental sample may have been produced by the microbiome more than 4 days before sampling if the environmental temperature is 20°C, or more than 15 days before if the temperature is 4°C. Thus, the time span during which one or more environmental parameters have affected microbial rRNA composition at the time of sampling varies with seasonal changes in temperature. It should be noted that our experimental set-up involved incubation at stable conditions. However, in most environments conditions are not stable and turnover rates *in situ* of microbial rRNA may be affected by daily or seasonal fluctuations in temperature, drought-induced lowering of soil water content, and other environmental fluctuations.

Our study involved a soil at low temperatures, but could be extended to unravel rRNA stability in other environments amenable to RNA preservation, e.g., environments with high osmolarity or low water activity. Precautions should be made when claims are made on the composition of the ‘active’ microbiome in these environments. Also, the stability of fungal and microeukaryotic rRNA awaits further studies.

In conclusion, the half-life of rRNA produced by a soil microbiome increased markedly with a decrease in environmentally relevant temperatures and was more than 7 months at −4°C. This means that rRNA may remain long after environmental conditions have changed and, thus, we should interpret with care correlations between rRNA-based microbiome structure and seasonal variation in ephemeral environmental parameters such as precipitation and concentration of soil water, redox potential, oxygen, inorganic nitrogen, and metabolomes.

## Data Availability Statement

All data generated during this study are included in this article and its Supplementary Material Additional File 1, which is a spreadsheet containing data from analyses of radioactivity and concentration of isolated RNA.

## Author Contributions

All authors designed the project, read, and approved the final manuscript. MS performed the experimental work. AP wrote the first draft of the manuscript with subsequent revisions performed by all authors.

## Conflict of Interest

The authors declare that the research was conducted in the absence of any commercial or financial relationships that could be construed as a potential conflict of interest.
